# Dystrophic Calcification of the Prostate after Cryotherapy

**DOI:** 10.1155/2014/471385

**Published:** 2014-12-07

**Authors:** Christopher Dru, Leon Bender

**Affiliations:** Cedars-Sinai Urology Academic Practice, Cedars-Sinai Medical Center, 8635 West 3rd Street, Suite 1070, Los Angeles, CA 90048, USA

## Abstract

We present a previously undocumented complication of dystrophic calcification of the prostate after cryotherapy. An 87-year-old male presented with recurrent lower urinary tract infections and was found to have an obstructing large calcified mass in the right lobe of the prostate. Subsequently, he underwent transurethral resection of the prostate (TURP) and bladder neck with laser lithotripsy to remove the calculus. We propose that chronic inflammation and necrosis of the prostate from cryotherapy resulted in dystrophic calcification of the prostate. As the use of cryotherapy for the treatment of localized prostate cancer continues to increase, it is important that clinicians be aware of this scenario and the technical challenges it poses.

## 1. Introduction

Dystrophic calcification occurs as a result of chronic inflammation or tissue necrosis. It is associated with several medical conditions such as collagen vascular disease, scleroderma, and systemic lupus erythematosis as well as with soft tissue injuries from trauma [1, 2]. In the genitourinary tract, dystrophic calcification has been reported in upper tract tumors, renal parenchymal disease, and squamous cell carcinoma of the bladder from schistosomiasis [3–5]. Cryotherapy of the prostate is a minimally invasive technique to treat localized prostate cancer. Under transrectal ultrasound (TRUS) guidance, transperineal probes are inserted into the prostate and cooled to −40°C resulting in tissue injury and coagulative necrosis. The most common side effects of cryotherapy are transient urinary retention from swelling, erectile dysfunction, and urethral sloughing [6]. However, there have been no reported cases of dystrophic calcification of the prostate in relation to cryotherapy.

## 2. Case Presentation

An 87-year-old male presented with recurrent episodes of hematuria and pelvic discomfort for six months due to recurrent lower urinary tract infections. He had a urological past medical history significant for low risk Gleason 3 + 3 prostate cancer (1/12 cores positive of right prostatic lobe only) treated with primary right-sided prostatic focal cryotherapy in 1996. His prostate cancer had been detected as a result of an elevated PSA and an abnormal digital rectal examination. At time of cryotherapy, he had no prostatic calcifications visible on TRUS or CT scan. Since his treatment, his serum PSA levels had been undetectable, and two subsequent CT scans of the abdomen and pelvis were negative for lymphadenopathy or evidence of metastatic disease. In addition, he had previously undergone a subtotal parathyroidectomy for hypercalcemia that had hence resolved before his diagnosis of prostate cancer.

**Figure 1:**
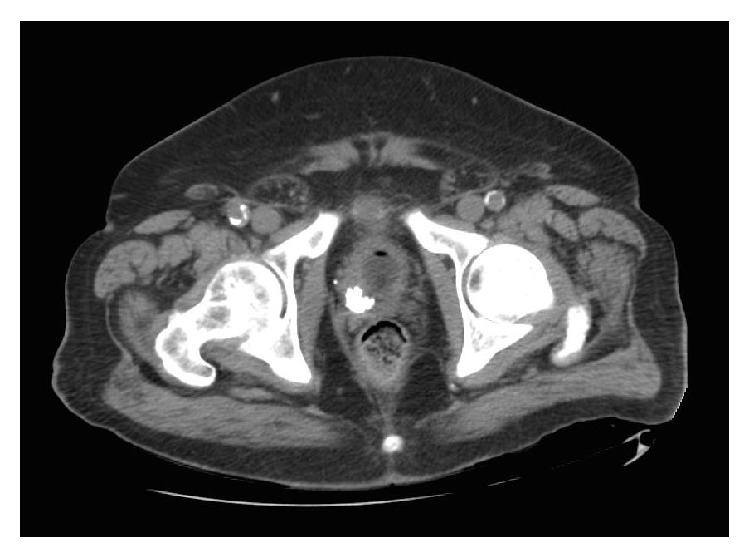
Axial image from the noncontrast pelvic CT scan demonstrating the right prostatic calcification measuring 17 × 12 mm in this section.

Physical examination of the patient revealed an alert and oriented male with minimal suprapubic tenderness. On digital rectal examination, the prostate was approximately 40 grams and smooth without irregularity. He had a 500 cc postvoid residual. The remainder of physical examination was within normal limits. Urine analysis was positive for leukocyte esterase and 26 WBC per HPF. His serum PSA was undetectable. A noncontrast CT of the abdomen and pelvis revealed bilateral renal cysts without hydronephrosis and a 17 × 15 × 12 mm calcification of the right lobe of the prostate (Figure 1).

The decision was made to proceed with TURP to relieve the obstruction as his symptoms had not improved with oral tamsulosin and finasteride. General anesthesia was induced, and a 22F cystoscope was passed through the urethra into the bladder without obvious signs of obstruction or trauma. Upon further investigation, there was enlargement of the right lateral lobe of the prostate from a protruding and visible calcified growth at the right bladder neck consistent with the previous CT findings. Lithotripsy with a holmium laser failed to penetrate the embedded stone given the intermixed soft tissue; therefore, a 27F resectoscope using a wedge loop was utilized to remove prostate and bladder neck tissue from around the stone. Once the soft tissue was removed, another attempt to laser the stone was performed; however, the laser failed to penetrate the stone. Again, the resectoscope using a wedge loop was utilized and relatively large sheets of stone were successfully removed with ease. A 24F three-way urinary catheter was left in place to gentle traction with continuous bladder irrigation overnight. The urine was clear of continuous bladder irrigation on postoperative day one, and the urinary catheter was removed. He was able to void freely with minimal postvoid residual and was discharged home that afternoon. Stone composition revealed calcium phosphate (hydroxy and carbonic apatite).

## 3. Discussion

Dystrophic calcification occurs in areas of chronic inflammation, tissue injury, and necrosis. This pathologic condition is best understood in relation to atherosclerosis and formation of atheromatous plaques in the aorta and other large arteries but is also well described in the rheumatologic and dermatologic literature [7, 8]. The pathogenesis involves an inciting event (nucleation) characterized by acute inflammation, followed by propagation and deposition of calcium phosphate crystals as chronic inflammation resolves. Tissue injury leads to calcification in two ways: leakage of calcium by damaged cell membranes becomes saturated to the point of crystallization and the acidic microenvironment from tissue necrosis inactivates endogenous calcification inhibitors [2, 9]. This process occurs in the corpora amylacea. Dystrophic calcification is independent of calcium and phosphate; however, high serum levels of these ions portend an increased risk of soft tissue calcifications [10]. Benign prostatic calcifications occur by a similar mechanism but on a much smaller level as most calcifications are <3 mm in greatest diameter and do not coalesce into large, symptomatic stones [11, 12].

In contrast to dystrophic calcification that occurs in injured tissue, metastatic calcifications occur in previously normal tissue. Metastatic calcifications are classified as either malignant or nonmalignant. As its name suggests, malignant metastatic calcifications occur as a result systemic or local malignancy—most notably parathyroid cancer, breast cancer, multiple myeloma, lymphoma, and leukemia [13, 14]. While the exact mechanism is not clearly understood, it is hypothesized that metastatic calcification deposits occur as a result of increased calcium metabolism from bone, induced by parathyroid hormone related protein (PTHrP) or alternations in expression of receptor activator of nuclear factor *κ*-*β* (RANK) and RANK ligand [15]. The elevated serum of levels of calcium ions can then precipitate in tissues that have a transient local-regional alkaline pH. It is impossible to predict where malignant metastatic calcifications will present and in which specific patients; however, calcifications are typically identified in the septa of the lungs, myocardium of the heart, muscular layer and gastric mucosa of the stomach, and the tubules of the kidneys [16]. Nonmalignant metastatic calcifications occur in a similar pattern of distribution but are attributed to benign causes such as hyperparathyroidism.

In the urology literature, benign prostatic calcifications have been described as a consequence of intermittent bouts of inflammation from recurrent urinary tract infections and prostatitis [17]. Additionally, inflammation from laser vaporization of the prostate for benign prostatic hyperplasia (BPH) is associated with symptomatic superficial bladder neck calcifications [18]. The mechanisms are likely similar given that inflammation is the commonality. In both of these reports, the calcifications typically presented as multiple, small, less than 2-3 mm calcifications, vastly different from the 1.7 cm calcification seen in our patient's prostate. Our patient had a remote history of hyperparathyroidism treated with subtotal parathyroidectomy years before he was diagnosed with prostate cancer. Since the time of treatment, his serum calcium levels had been normal, and there were no documented prostatic calcifications before his cryotherapy, ruling out nonmalignant calcification as the cause of his prostatic calcification. Additionally, from the time of the diagnosis of prostate cancer until present, there was no evidence to suggest that the patient had any forms of solid organ or systemic malignancy based upon laboratory findings, clinical imaging, and physical exam. Dystrophic calcification of the prostate is the most likely etiology of the patient's prostate stone as (1) the right prostatic calcification occurred in the area of previous tissue injury and necrosis consistent with the location of right-sided prostatic focal cryotherapy the pathogenesis of dystrophic calcification, (2) there was no evidence of calcification of any area of the prostate before cryotherapy, and (3) there is no evidence to support any type of metastatic calcification as the cause.

Early reports of surgical cryotherapy date back to the late 1800s when cervical and breast masses were treated with mixtures of salt and ice to reduce tumor volume and treat local symptoms [19]. In the years to come, the technology improved. In 1961, the first liquid nitrogen cryotherapy probe system was developed by Cooper and Lee, which allowed for targeted freezing of tissue to −200 degrees Celsius [20]. The technology was adopted by urologists Soanes and Flocks to treat prostate cancer and BPH with transperineal cryotherapy through visual observation of tissue freezing; however, given inadequate monitoring capabilities, many patients became incontinent or developed fistulas [21, 22]. The advent of a urethral warming catheter in the 1990s helped to prevent these devastating complications but other surgical modalities, such as open and robotic-assisted radical prostatectomy, brachytherapy, and radiation for the treatment of prostate cancer prevailed [23]. Now, in the 21st century, with more emphasis on minimally invasive techniques and the development of real-time ultrasound-guided imaging, cryotherapy of the prostate has made resurgence as an acceptable alternative in the management of localized prostate cancer.

Primary cryotherapy for the treatment of prostate cancer is an option for men with T1-2 N0M0 localized disease who have not previously undergone TURP [24]. Ideal candidates should not have large prostates, as this inhibits the ability to uniformly achieve cold temperatures throughout the entire gland. Liquid nitrogen has been replaced with argon as the cooling medium. Percutaneous perineal probe placement is performed under TRUS guidance; unilateral or bilateral prostate templates can be utilized [25]. The five-year biochemical disease-free survival rates are 65–92%, 69–89%, and 48–89% for low-, intermediate-, and high-risk cases of prostate cancer [26]. Additionally, salvage cryotherapy is a treatment option for patients who fail prostate external beam radiation therapy with recurrent focal disease.

Repeat imaging is not part of the standard follow-up care after cryotherapy of the prostate and is only performed if a patient has a persistently elevated prostate specific antigen or abnormality on digit rectal examination. Therefore, it is not known what percentage of patients develop sizeable calcifications of the prostate after cryotherapy. In this patient, the calcification protruded through the prostate and into the bladder neck causing bladder outlet obstruction and recurrent urinary tract infections. This suggests that the cellular damage caused by the cryotherapy probe extended well into the bladder neck to cause coagulation necrosis and later dystrophic calcification. As the use of cryotherapy for the treatment of localized prostate cancer continues to increase, it is important that clinicians are aware of this potential scenario and the technical challenges it poses.
